# Supplementary material for: Effect and feasibility of district level scale up of maternal, newborn and child health interventions in Pakistan: a quasi-experimental study
Source: BMJ Open. 2020 Jul 14;10(7):e036293. doi: 10.1136/bmjopen-2019-036293 (PMC7365487; doi:10.1136/bmjopen-2019-036293)
Supplement: Author's manuscript [file bmjopen-2019-036293.draft_revisions.pdf]

BMJ Open

BMJ Open is committed to open peer review. As part of this commitment we make the peer review history of every article we publish publicly available.

When an article is published we post the peer reviewers' comments and the authors' responses online. We also post the versions of the paper that were used during peer review. These are the versions that the peer review comments apply to.

The versions of the paper that follow are the versions that were submitted during the peer review process. They are not the versions of record or the final published versions. They should not be cited or distributed as the published version of this manuscript.

BMJ Open is an open access journal and the full, final, typeset and author-corrected version of record of the manuscript is available on our site with no access controls, subscription charges or pay-per-view fees (http://bmjopen.bmj.com).

If you have any questions on BMJ Open's open peer review process please email info.bmjopen@bmj.com

Implementing effective maternal, newborn and child health interventions in Pakistan: a protocol for the Umeed-e-Nau (New Hope) district-level scale-up project

Journal:	*BMJ Open*
Manuscript ID	bmjopen-2019-036293
Article Type:	Protocol
Date Submitted by the Author:	10-Dec-2019

Complete List of Authors:	Memon, Zahid Ali; Aga Khan University, Centre of Excellence in Women and Child Health<br>Muhammad , Shah; Aga Khan University, Centre of Excellence in Women and Child Health<br>Soofi, Sajid; Aga Khan University Hospital, Paediatrics<br>Khan , Nimra ; Aga Khan University, Centre of Excellence in Women and Child Health<br>Akseer, Nadia; The Hospital for Sick Children , Centre for Global Child Health<br>Habib, Atif; Aga Khan University, Department of Paediatrics & Child Health<br>Bhutta, Zulfiqar; Aga Khan University, Division of Women and Child Health; The Hospital for Sick Children , Centre for Global Child Health

Keywords:	Community child health < PAEDIATRICS, Quality in health care < HEALTH SERVICES ADMINISTRATION & MANAGEMENT, PUBLIC HEALTH

SCHOLARONE™
Manuscripts

I, the Submitting Author has the right to grant and does grant on behalf of all authors of the Work (as defined in the below author licence), an exclusive licence and/or a non-exclusive licence for contributions from authors who are: i) UK Crown employees; ii) where BMJ has agreed a CC-BY licence shall apply, and/or iii) in accordance with the terms applicable for US Federal Government officers or employees acting as part of their official duties; on a worldwide, perpetual, irrevocable, royalty-free basis to BMJ Publishing Group Ltd ("BMJ") its licensees and where the relevant Journal is co-owned by BMJ to the co-owners of the Journal, to publish the Work in this journal and any other BMJ products and to exploit all rights, as set out in our licence.

The Submitting Author accepts and understands that any supply made under these terms is made by BMJ to the Submitting Author unless you are acting as an employee on behalf of your employer or a postgraduate student of an affiliated institution which is paying any applicable article publishing charge ("APC") for Open Access articles. Where the Submitting Author wishes to make the Work available on an Open Access basis (and intends to pay the relevant APC), the terms of reuse of such Open Access shall be governed by a Creative Commons licence – details of these licences and which Creative Commons licence will apply to this Work are set out in our licence referred to above.

Other than as permitted in any relevant BMJ Author's Self Archiving Policies, I confirm this Work has not been accepted for publication elsewhere, is not being considered for publication elsewhere and does not duplicate material already published. I confirm all authors consent to publication of this Work and authorise the granting of this licence.

For peer review only

Implementing effective maternal, newborn and child health interventions in Pakistan: a protocol for the Umeed-e-Nau (New Hope) district-level scale up project

Zahid A Memon, MPH [1]; Shah Muhammad, MPH [1]; Sajid B Soofi, FCPS[1] Nimra Khan, MPH [1]; Nadia Akseer PhD [2]; Atif Habib, PhD[1]; Zulfiqar A Bhutta PhD [1, 2]

[1]Center of Excellence in Women and Child Health, Agha Khan University, Karachi, Pakistan
[2] Centre for Global Child Health, The Hospital for Sick Children, Toronto, ON, Canada

Corresponding Author:
Zulfiqar A. Bhutta
Qualification: FRCPCH, PhD
Founding Director, Center of Excellence in Women and Child Health
Aga Khan University, zulfiqar.bhutta@aku.edu
+92214930051 ext. 4782
Centre for Global Child Health, The Hospital for Sick Children, 686 Bay St, Ste 11.9801, Toronto, ON M5G 0A4, Canada (zulfiqar.bhutta@sickkids.ca).

For peer review only

ABSTRACT

Introduction

Pakistan has a high burden of maternal, newborn and child morbidity and mortality. Several factors including weak scale-up of evidence-based interventions within the existing health system; lack of community awareness regarding health conditions; and poverty contribute to poor outcomes. Deaths and morbidity are largely preventable if a combination of community and facility-based interventions are rolled out at scale.

Methods and analysis

The overall aim of the Umeed-e-Nau (UeN) project is to improve MNCH in 8 high burden districts of Pakistan by scaling up of evidence-based interventions. The project will assess interventions including 1) Developing job aids and training material; 2) Building Capacity of providers and workers; 3) Ensure Provision of essential MNCH commodities; 4) Revitalization of community women support groups and village health committees and deployment of community health workers in areas which do not have LHWs; 5) Promote use of routine service delivery data for decision making; 6) Engage stakeholders.

We hypothesize that roll out of the UeN project will lead to a significant reduction in perinatal mortality rate (20% compared to baseline) and at least 30% reduction in diarrhea and pneumonia case fatality rates in the target districts where two intervention groups will serve as internal controls.

Household surveys will be conducted at three points (baseline, midline, and endline) to track progress and assess the impact of the UeN project. Descriptive and multivariate methods will be used for assessing the association between different factors and a difference in difference estimates will be used to assess the impact of the intervention on outcomes.

Ethics and Dissemination

The ethics approval of the study was obtained from the Aga Khan University Ethics Review Committee on February 28th, 2017.

This trail is registered, NCT04184544.

Key Words

Maternal, Neonatal, Child Health, Interventions, Mortality, Community child health, Public Health, Quality of care

Word Count 3669

For peer review only

Strengths and limitations of this study

- Strength of the UeN project includes its quasi experimental study design with district representative sample size
- Intervention packages encompassing strengthening of the public health facilities; community outreach program using existing platforms and private sector engagement strategy will be assessed in Pakistan for the first time
- Evaluation of an intervention to improve quality of all sources of routine service delivery data to promote decision making has never before been assessed in Pakistan
- Potential limitations might relate to the reluctance of various health sector cadres particularly private sector to provide routine service delivery data for monitoring and evaluation purposes
- Challenges of working with or assessing role of LHWs who are already overburdened with competing priorities, organizations running public health facilities under public private partnership initiative and data quality issues in routine service delivery data.

For peer review only

INTRODUCTION

Pakistan's high maternal, neonatal, and child mortality continues to hamper its overall growth and development.[1] The global maternal mortality ratio in 2017 is estimated at 211 maternal deaths per 100 000 live births while 2019 estimates by United Nations inter-agency group for child mortality estimation (UN-IGME) estimate neonatal mortality rate (NMR) at 18 deaths per 1000 live births and under-5 mortality rate is 39 per 1,000 live births per year globally.[2,3] Diarrhea and pneumonia are two of the most common causes of child mortality; together, they account for more than 30% of all under-5 deaths in Pakistan.[4]

Several factors have contributed to the slow progress in maternal, newborn, and child health (MNCH) in Pakistan, including social determinants such as poverty, high rates of illiteracy, lack of women's and girls' empowerment, voice, and high fertility rates. These are compounded by poorly functional health systems with low coverage rates of essential health interventions, wide disparities by geographic and socio-economic status and a largely unregulated private sector providing poor quality health services;[5] and lack of community awareness and timely care seeking for health conditions.[6,7,8] The majority of maternal, newborn and child deaths are preventable and treatable given programs are designed and delivered through an efficient public and private health system that takes into account the geographic and social structures.[9]

A number of strategies have been employed at community and facility levels to strengthen the public health service delivery system in Pakistan and elsewhere.[10] Such approaches rely on delivery of preventive as well as basic curative services through community-based healthcare workers and providers with improved and effective referral pathway.[11] For example, a low cost community-based intervention package within the health system settings in Gilgit region of Pakistan had a significant reduction of 38% in perinatal mortality rate.[12] The maternal and newborn intervention project conducted in Hala, Pakistan through community health workers resulted in 21% reduction of in stillbirth rate and 15% neonatal mortality rate over two years period.[13] Community management of pneumonia through LHWs showed LHWs can successfully diagnose and treat severe pneumonia at home. The LHWs were able to effectively reach children with pneumonia where referral was difficult.[11] Application of 4% chlorhexidine to the umbilical cord to prevent omphalitis and thereby effecting neonatal mortality was piloted resulting in 38% reduction in neonatal mortality.[14] A diarrhea pack study was feasible and acceptable with an impact on the incidence of diarrhea and reduced hospitalization rates.[15] Community

For peer review only - http://bmjopen.bmj.com/site/about/guidelines.xhtml

case management of pneumonia through LHWs with oral amoxicillin at home was tested in two districts of Pakistan and was proven to be safe and effective for treatment of severe pneumonia through LHWs.[16] Furthermore, there is evidence on interventions for strengthening health information system.[17]

Lastly, it is evident from previous studies that scaling up interventions using delivery platforms to reach poor and rural populations through community-based strategies and their successful implementation can prevent 58% of an estimated 367 900 deaths (15 900 maternal, 169 000 newborn, 183 000 child deaths) and 49% of an estimated 180 000 stillbirths in Pakistan alone.[11,12,13, 14, 15, 17,18]

The UeN initiative aims to improve the health, well-being and survival of women and children in Pakistan through piloting and implementing effective MNCH interventions at scale in 8 high burden districts spanning 3 provinces and targeted to reach more than 11 million beneficiaries. The UeN initiative's main goal is to scale up the evidence-based and cost-effective MNCH interventions within existing platforms at the community and facility level. Study goal, objectives and activities are detailed in Figure 1. It is anticipated the UeN intervention scale up in 8 districts will reach approximately 1.7 million under-5 children, 1.5 million women of reproductive age, and 8.6 million other civilians. The intervention will target 509 public health facilities (19 maternal and child health centers, 150 Government dispensaries, 255BHUs, 59 RHCs, 18 THQs 6 DHQs, 2 civil hospitals), 500 private providers, 6518 LHWs, and 300 CHWs.

This protocol paper will present the design and roll-out strategy for the UeN initiative. We will outline the setting, methods, implementation strategies and monitoring & evaluation processes of the project roll-out spanning years 2017-2022 (project timeline shown in Figure 2).

METHODS AND ANALYSIS

Study setting

Covering 3 provinces in the country, the UeN scale up component will be implemented in 8 districts (location of interventions by theme is shown in Figure 3). Study districts were selected to be predominantly rural, and had some of the highest MNCH morbidity and mortality outcomes in the country. An important consideration was also ensuring that selected districts did not have other major public health campaigns to avoid conflation or dilution of UeN impact. High burden districts were strategically selected to demonstrate effective change models in difficult geographies so as to maximize project impact on population outcomes. Table 1 [19] outlines relevant demographic, health and contextual indicators for each study district.

For peer review only - http://bmjopen.bmj.com/site/about/guidelines.xhtml

Table 1: Distribution of demographic and health indicators for maternal, newborn and child health indicators in districts selected for program implementation in UeN initiative in Pakistan

	Maternal and Newborn Health		Maternal health	Newborn Health	Child Health			
	**Lasbela**	**Badin**	**Sanghar**	**Nasirabad**	**Qambar Shahdadkot**	**Muzaffargarh**	**Jafferabad**	**Rahim Yar Khan**
**Population**	574,292	1,804,516	2,057,057	490,538	1,341,042	4,325,483	513,813	4,814,006
**U5 population**	74,657	202,106	230,390	13,837	150,197	462,756	14,191	625,8208
**Human development index (HDI)**	0.416	0.412	0.491	0.311	0.456	0.584	0.345	0.625
**HDI rank**	85	87	72	98	77	58	94	51
**Tehsils/Talukas**	5	5	6	4	7	4	3	4
**Union Councils**	22	12	63	31	52	93	38	139
**Vaccination coverage- Children 12-23 months (fully immunized)**	28.8	45.5	47.4	28.8	25.6	82.1	28.8	72.0
**ANC during last pregnancy (%)**	55.9	83.1	56.8	55.9	64.5	37.4	55.9	30.8
**Facility births (%)**	34.6	64.8	40.0	34.6	48.7	50.7	34.6	62.0
**Delivery by Skilled Birth Attendant**	38.2	66.6	42.1	38.2	49.7	52.7	38.2	65.6
**Postnatal consultation in Health Facility**	29	75.0	61.6	29	68.2	56.9	29	63.9

For peer review only - http://bmjopen.bmj.com/site/about/guidelines.xhtml

**Access to tap water**	29.6	20.7	20.7	28.6	5.8	6.5	28.6	21.4
**Access to toilet (Flush)**	17.6	70	51.8	16.5	77.3	72	16.5	60.5
**Number of LHWs**	153	1010	883	309	680	1891	130	1723
**LHWs coverage %**	50	80	66	60	59	51	50	80
**Number of Hospitals**	125	145	116	97	271	248	97	260
**Number of RHCs**	4	12	5	1	4	12	1	19
**Number of BHUs**	42	36	58	19	4	76	19	103

Study design

In order to evaluate the impact of scaling up MNCH interventions, a quasi-experimental study design will be used. Each of the 8 districts will be treated as an intervention site, and will be grouped together broadly into two areas to span MNCH interventions (Figure 3). As detailed below, group 1 will receive maternal and newborn health (MNH) interventions, while group 2 will receive child health (CH) interventions. Each group will serve as the "control" for the other. This design will help us evaluate independent and combined effect of maternal, newborn, and child health interventions to address MNCH outcomes.

Site allocation to Group1 and 2 were done through extensive consultation with provincial government, bilateral donors and UN agencies, and UeN project team. Factors that were considered during intervention allocation included level of MNCH morbidity and mortality, assuring geographical diversity across the provinces, varying size of the districts, and government MNCH priorities in those districts(i.e. so that those areas that were focused on improving CH, for example, received the CH interventions).

For peer review only - http://bmjopen.bmj.com/site/about/guidelines.xhtml

Sample size

The sample size for the household surveys will be calculated using the assumption that that the roll out of proven and effective interventions within existing health delivery platforms for MNCH will lead to significant reduction in perinatal mortality rate (20% compared to baseline) and at least 30% reduction in diarrhea and pneumonia case fatality rates in the target districts for the proposed UeN scale-up initiative. Perinatal mortality will be used as the primary outcome for all sample size projections. A stratified two stage cluster sampling approach will be used to calculate sample size. Moreover, it is important to power the study to provide a detectable difference between the two groups of Maternal and Newborn health group, and the Child Health group. We will use a before and after design for sample size calculation. For maternal and newborn health districts, assuming a 20% reduction in the Perinatal Mortality (PNM) from the baseline PNM of 75 per 1000 live births (Pakistan Demographic Health Survey 2012-13 for rural Pakistan)[20] with a power of 80% to detect the difference in the PNM between baseline, and the end line household survey, a type I error of 5%, design effect of 1.515 and an annual attrition of 10% per year, at least 19,582 households will be enrolled in the survey. For Child Health intervention districts, assuming a 30% reduction in the Post Neonatal Death (PND) rate of 19/1000 live births at baseline (Pakistan Demographic Health Survey 2012-13 for rural Pakistan) with a power of 80% to detect the difference in the PND between baseline, and the end line household survey, a type I error of 5%, design effect of 1.388 and an annual attrition of 10% per year, at least 19,210 households have to be enrolled in the survey.

Intervention packages and implementation strategies

An integrated intervention package, that has shown to be relevant, feasible, and effective is designed thematically (Figure 4) and will be delivered at scale in the target districts. Group 1: Maternal Health: These 4 districts (Badin, Sanghar, Lasbela, Nasirabad) will receive core MNH interventions as included in global recommendations for maternal health (Helping Mothers Survive)[21] and newborns (Helping Babies Survive)[22] consisting of three modules: Helping Babies Breathe, Essential Care for Every Baby, and Essential Care for Small Babies. We stratified intervention delivery into 3 sub-groups focused separately on maternal health (1 district: Sanghar); newborn health (1 district: Nasirabad); and combined maternal and newborn health (two districts: Lasbela and Badin). This stratification was undertaken to independently evaluate the relative impact of theme-specific interventions on key MNH and mortality outcome indicators; i.e. akin to a 3-arm trial design.

For peer review only - http://bmjopen.bmj.com/site/about/guidelines.xhtml

Group 2: Child Health: These 4 districts (Qambar Shahdadkot, Muzaffargarh, Rahim Yar Khan and Jafferabad)representing 3 provinces will receive child health interventions focusing on the implementation of the Global Action Plan for Pneumonia and Diarrhea (GAPPD).[23]

Details on UeN intervention approaches packages are displayed in Figure 1 and are discussed below by each of the 5 objectives.

Objective 1: Improve Quality of MNCH Care

Activity 1.1. Development of training material and job aids

UeN will adopt and contextualize development of training material and job aids in accordance with global and national guidelines to build provider knowledge, skills, and practices. The project team along with experts from the academia and government programs will develop training packages (including trainer and learner materials, as well as supplemental job aids and guidance) for outreach community health workers and for public and private HCPs.

For private HCPs, 14 modules for on-site learning using academic detailing to refresh the private providers' knowledge will be developed.

Activity 1.2. Capacity Building

UeN will employ a mix of capacity building activities to address both outreach workers' and public sector HCPs' performance and confidence in achieving quality standards. Training content will be based on the district's theme. Topics to be covered in the training are listed in Figure 4.

- *Outreach Health Workers Training: Lady Health Workers*

The LHWs' skills will be strengthened to promote healthy MNCH practices and behaviors at community-level and will be trained to adhere to care management protocols and use new commodities and technology which includes chlorhexidine, amoxicillin dt, zinc dt and low osmolality ORS and respiratory counters etc. The LHW training will be based on low dose high frequency model. [12,13]

- *Facility based: Training for providers at primary and secondary level public health facility*

Trainings for public sector HCPs will be group-based trainings where participants will gather in a public health care facility for a competency-based learning activity.

Activity 1.3. Private sector engagement

Private sector HCPs will be engaged through academic detailing, where individual health providers from private sector (or a facility-based team of providers) will participate in a structured learning activity at their facility. Topics on key areas of MNCH service delivery will be covered.

For peer review only - http://bmjopen.bmj.com/site/about/guidelines.xhtml

Initial interaction will be followed by facility-based coaching and mentoring.

Activity 1.4. Sustained key commodities and supplies by increasing efficiency of the supply chain for essential commodities at community and facility level

Stock out of MNCH commodities is a major bottleneck in delivering quality services within the health system.[24] Community outreach workers, primary and secondary level health care facilities in the target districts will be equipped with essential drugs and equipment to provide quality services through engaging officials of the department of health and other stakeholders. The commodities and supplies will be ensured according to district themes such as for maternal and newborn health districts, clean delivery kit will be provided to LHWs.

For the four MNH districts, clean delivery kits will be provided by AKU. For the CH districts, supplies of amoxicillin dt (250 mg), zinc dt, ARI timer and pulse oximeter will be provided by UNICEF in three districts of Punjab and Sindh province, however since the current UNICEF programme does not cover provision of commodities to Balochistan, AKU will supply the commodities to the child health district in Balochistan.

For peer review only

Objective 2: Improve access to and uptake of MNCH services through strengthening supply and demand

Activity 2.1. Increase in Demand

- **Improve uptake of healthy practices at household level through community mobilization**

Through community mobilization activities the community will be informed through LHWs about danger signs during pregnancy, child birth, infancy and childhood. The importance of early referral by LHWs to health facilities for prevention of morbidity and mortality will be the focus of community mobilization activities.

- **Community engagement through revitalization/creation of women support groups/ village health committees**

LHWs will be trained to revitalize and create where required the self-help groups, village health committees and women support groups to discuss MNCH issues. These groups are expected to help women to adopt healthy MNCH practices.

- **Expanding the coverage to underserved areas**

In current public health outreach system, LHWs provide services at the household level. However, only 50-60% of the rural districts are covered by LHWs.[25] To serve the geographic areas for which LHWs are not appointed, CHWs will be hired and will be trained on technical MNCH content, registration, community support group methodology and village health committee formation. Around 100 per district will be deployed. CHWs will be selected on the lower education qualification such as primary education or lower as compared to LHWs who are minimum middle pass. The CHW training will consist of lectures, interactive discussions, and role-plays to develop the skills of performing their roles. The duration of the training will be two weeks, twice in the first year, and then a refresher training every year. CHWs will only be recruited and deployed in districts that have maternal, newborn or combined component due to current scope of community health worker programs.

Objective 3: Promote use of data for decision-making

In Pakistan, there are two main sources of data for decision making the District Health Information System (DHIS) and the LHW-Management Information System (MIS) for health facility and community level information respectively. The private sector HCPs do not share routine service delivery data in the DHIS. To promote data use; UeN's objective is to target all three data sources. Initially, a gaps analysis will be conducted to identify the weaknesses in the system

Activity 3.1. Improving public health facility related information system i.e. DHIS

The routine service delivery data of both primary and secondary level health facilities is managed through DHIS system. The DHIS collects data on the 18 priority diseases, availability of drugs, service utilization

For peer review only

and human resource of health facilities. Data from each district is discussed in the quarterly district level meetings. However, there is poor compliance with the source registers which is the main source of information. As the data quality is poor and questionable, therefore it cannot guide decision making.[26]To improve understanding and importance of collecting data; HCPs of public health facilities will be trained on collection, recording and reporting of the data for DHIS.

For better compliance with source register and data quality, the project team will collect data from source registers on 40 key MNCH indicators every month. Data for these indicators will also be extracted from DHIS monthly reports from the online available dashboard. Values for the indicators from both source register and DHIS dashboard will be compared to assess data completeness and quality.[27] Monthly feedback report on data quality and completeness will be shared with the managers of health facilities and district managers. Furthermore, quarterly feedback report will be provided about the data quality and summary measures of key MNCH indicators in the district level management meetings.

Activity 3.2 Improving community based health information system i.e. LHW-MIS

The LHW is affiliated with a primary health facility and has a catchment population of 1000 and maintains their record e.g. number of married couple, pregnant women, under-5 children and service provision on source registers provided by the LHW program.[25] The information collected from the community level, is compiled on a monthly report and shared with their supervisors LHS and then relayed to the district coordinator for the LHW Program. However, the data quality is not monitored regularly and data use for decision-making is minimal. To improve the data quality, the LHS and LHWs will be trained on the importance of accurate data recording and reporting. Furthermore, 10 LHWs will be randomly selected from their reporting health facility every month; data from their source registers will be collected on 29 MNCH related indicators on an android application.

Values for the indicators from both source register and LHW-MIS dashboard will be compared to assess data completeness and quality. Monthly feedback reports will be shared with LHS and LHWs. Furthermore, quarterly feedback report about the data quality and summary of key MNCH indicators will be shared in the district level meetings.

Activity 3.3.Establishing Private Providers –Management Information System

A private provider – management information system will be established. Routine data collection registers similar to the DHIS source registers with indicators according to the district themes will be developed and distributed to the private providers. Private providers will be encouraged to record data. The project team will collect data on monthly visits from the health facility. This will enable capture of MNCH related morbidities and mortalities which are otherwise lost due to non-reporting.

For peer review only - http://bmjopen.bmj.com/site/about/guidelines.xhtml

Objective 4: Encourage Advocacy and Partnerships

Stakeholder engagement activities will be conducted at different levels to inform policy, community, and district health authorities to garner support for the UeN initiative.

Activity 4.1. Participation in provincial child survival technical groups

For reducing Diarrhea and Pneumonia incidence, UNICEF has initiated the establishment of provincial child survival committees in which UeN will participate. There are also existing provincial MNCH committees where AKU will have a seat by virtue of existing projects and the current UeN project. The UeN project will set up a system of monthly meetings with UNICEF.

Activity 4.2. Formation of Project Advisory Committee at National level

Moreover, National ministry of health services regulation and coordination will be requested to constitute a project advisory committee to help guide the UeN project through the existing federal ministry of health services and regulation to ensure coordination. The committee will meet once during the inception phase to discuss the project structure and remit, followed by bi-annual progress reviews to address discuss challenges in implementation and address with appropriate actions.

Objective 5: Monitoring and Impact Evaluation

UeN scale up will undertake rigorous monitoring and evaluation of core activities to ensure project progress and achievement of desired goals. Specific activities are detailed below:

Activity 5.1. Household surveys

Household surveys will be conducted at three time points, baseline, at approximate mid-line when the interventions have been implemented at scale for at least 18-24 months, and at the end of the project (towards the end of year 5). The surveys will collect information to assess the impact of the interventions on primary and secondary outcome indicators (listed in table 2).

Table 2: List of primary and secondary outcomes

**Outcomes**	**Definition**
**Primary Outcomes**	
**Perinatal mortality rate**	The number of stillbirths and deaths in the first week of life per 1,000 total births
**Diarrhea case fatality**	Percent deaths among under-5 children due to diarrhea

For peer review only - http://bmjopen.bmj.com/site/about/guidelines.xhtml

**Pneumonia case fatality**	Percent deaths among under-5 children due to Pneumonia
**Secondary Outcomes**	
**Maternal and Newborn Health**	
**Contraceptive use**	Percent of mothers aged 15-49 who are using modern contraceptive method
**Antenatal care coverage**	Percent of mothers aged 15-49 of who received antenatal care ≥4 times during pregnancy with their youngest living child aged <6 months
**Tetanus Toxoid coverage**	Percent of mothers aged 15-49 who received ≥2 doses of tetanus toxoid during pregnancy with their youngest living child
**Skilled birth Attendant at time delivery**	Percent of mothers aged 15-49 whose youngest living child was delivered by skilled health personnel
**Institutional Delivery**	Percent of mothers aged 15-49 whose youngest living child was delivered in a health facility
**Postnatal care coverage**	Percent of mothers aged 15-49 who received postnatal care (for self or for infant) within 3 days of birth
**Early initiation of breastfeeding**	Percent of mothers aged 15-49 whose youngest living child was breastfed within one hour of birth
**Exclusive breastfeeding**	Percent of mothers aged 15-49 whose youngest living child was exclusively breastfed
**Vaccination coverage among under-5 children**	Percent of mothers aged 15-49 whose youngest living child received ≥3 doses of DPT/pentavalent vaccine when aged 12-23 months

For peer review only - http://bmjopen.bmj.com/site/about/guidelines.xhtml

	Percent of mothers aged 15-49 whose youngest living child received ≥1 dose of measles vaccine when aged 12-23 months
	Percent of mothers aged 15-49 whose youngest living child was fully immunized
**Care-seeking for Diarrhea among under-5 children**	Percent of mothers aged 15-49 whose youngest living child with diarrhea in the previous 2 weeks received ORS and zinc
**Care-seeking for Pneumonia among under-5 children**	Percent of mothers aged 15-49 whose youngest living child with pneumonia-like symptoms in the previous 2 weeks was taken to an appropriate health provider and received antibiotics
**Capacity building**	Number of healthcare providers trained per 10 000 population
	Number of LHWs trained per 10 000 population

Activity 5.2. Quality of care assessments

Health facility assessment will be conducted bi-annually to provide information on facility level performance and service quality including availability of commodities and supplies, human resource and key equipment as well as observation of HCPs will be done using quality of care checklists to assess the effectiveness of trainings provided. In case of inadequate availability of resources or sub-optimal quality of care provided, arrangements for supplies will be done by liaising with stakeholders and refresher trainings will be provided to HCPs. All public health facilities (509 including 19 Maternal and child health centers, 150 Government dispensaries, 255BHUs, 59 RHCs, 18 THQs 6 DHQs, 2 civil hospitals) will be included in the study. For private providers, all health practitioners who provide services according to district theme will mapped. Practitioners with high patient flow will be included in the study for quality assessment and needs based support will be ensured.

Activity 5.3. Assessment of the community outreach program (i.e. LHW program)

For peer review only - http://bmjopen.bmj.com/site/about/guidelines.xhtml

The activities of LHWs will be monitored on regular basis to strengthen monitoring system of LHW program and improve LHW functionality. 30% health facilities will be selected on quarterly basis, 10 LHWs will be selected using purposive sampling from each health facility. Data will be recorded in mobile application from the LHW's source registers for community support groups and 10 randomly selected households (*khandans*). The data for community support groups will be validated from the community and households will be visited to validate the household level data.

Study outcome measures

The primary outcomes of interest are reduction in perinatal mortality rate (20% compared to baseline) and at least 30% reduction in Diarrhea and Pneumonia case fatality rates. The secondary outcomes include coverage of essential intervention coverage across the continuum of care and maternal, newborn and child morbidity rates (Table 2).

Analysis Plan

Data from baseline survey will be analyzed at two levels; one during the data collection to provide information for monitoring the progress and identify any errors in data collection. The second phase includes using software to analyse data. This will only begin when it is ensured that data is clean. The second phase will be more advance analysis.

Data will be analysed using univariate and multivariate methods. Descriptive statistics for the subjects will be obtained and Pearson Chi square test will be used to establish association between categorical variables. Simple frequency tables will be generated to ascertain the information on socio-economic and demographic data. Data analysis will be conducted based on the agreed dummy tables.

Further Multivariate analysis will be done to establish a true association between the factors that will be found to be significant during the bivariate analysis. To ascertain the adjusted odds ratio controlling for confounding factors we will use Backward LR model to establish the association. Various factors will be considered as independent variable while the outcome measures that will found significant on bivariate analysis will be considered as dependent variables.

Assuming parallel trends within the two MNH and CH groups, in the absence of any intervention, difference-in-difference estimates will be used to assess the impact of the intervention on outcomes. An interaction term between group (intervention/control) and survey wave (baseline/end-line) will be included in each model. All the analyses will be done using STATA.

For peer review only - http://bmjopen.bmj.com/site/about/guidelines.xhtml

Ethics:

Ethics approval of the study was from obtained from the Aga Khan University Ethics Review Committee on February 28th, 2017. In additions, ethical clearance was sought from National Bioethics committee (NBC), Pakistan.

Patient and Public Involvement:

The public was not involved in the design of the research tools but they will be part of the study and their feedback will be sought regarding intervention acceptability. The key findings will be shared with their representatives as part of the dissemination plan at local level.

Authors' contributions:

ZAB conceived the project and provided the technical and intellectual inputs as principal investigator in writing this manuscript and approved for submission. ZM produced the first draft and subsequent drafts of the paper. NA, NK, SM and SS were involved in review and provided inputs on various aspects of the manuscript. All authors reviewed and approved various drafts and the final paper.

Funding statement: This work is supported by Bill and Melinda Gates Foundation grant number [OPP1148892].

Acknowledgments: We thank Rahat Qureshi, Shabina Arif, Ali Turab and their respective teams for their support in designing the intervention packages around maternal, newborn and child health.

Competing interests: The authors declare that they have no competing interests.

For peer review only

References

1. Bhutta ZA, Hafeez A. What can Pakistan do to address maternal and child health over the next decade? *J Health research policy and systems* 2015;13(1):S49.
2. World Health Organisation. Trends in Maternal Mortality: 1990 to 2019 Estimates by WHO, UNICEF, UNFPA, World Bank Group and the United Nations Population Division. Geneva: World Health Organisation, 2019.
3. United Nations inter-agency Group. Levels & Trends in Child Mortality Report 2019: Estimates Developed by the UN Inter-agency Group for Child Mortality Estimation, 2019.
4. Bhutta ZA, Hafeez A, Rizvi A, et al. Reproductive, maternal, newborn, and child health in Pakistan: challenges and opportunities. *The Lancet* 2013;381(9884):2207-18.
5. Shaikh BT. Private sector in health care delivery: a reality and a challenge in Pakistan. *Journal of Ayub Medical College Abbottabad* 2015;27(2):496-98.
6. Kassebaum NJ, Bertozzi-Villa A, Coggeshall MS, et al. Global, regional, and national levels and causes of maternal mortality during 1990–2013: a systematic analysis for the Global Burden of Disease Study 2013. *The Lancet* 2014;384(9947):980-1004.
7. World Health Organisation. WHO guidelines on preventing early pregnancy and poor reproductive outcomes among adolescents in developing countries Geneva: World Health Organisation 2011.
8. Khan NN, Puthussery S. Stakeholder perspectives on public-private partnership in health service delivery in Sindh province of Pakistan: a qualitative study. *J Public health* 2019;170:1-9.
9. Bhutta ZA, Cabral S, Chan Cw, et al. Reducing maternal, newborn, and infant mortality globally: an integrated action agenda. *J International Journal of Gynecology Obstetrics* 2012;119:S13-S17.
10. Bhutta ZA, Black RE. Global maternal, newborn, and child health—so near and yet so far. *J New England Journal of Medicine* 2013;369(23):2226-35.
11. Soofi S, Ahmed S, Fox MP, et al. Effectiveness of community case management of severe pneumonia with oral amoxicillin in children aged 2–59 months in Matiari district, rural Pakistan: a cluster-randomised controlled trial. *The Lancet* 2012;379(9817):729-37.
12. Memon ZA, Khan GN, Soofi SB, et al. Impact of a community-based perinatal and newborn preventive care package on perinatal and neonatal mortality in a remote mountainous district in Northern Pakistan. *J BMC pregnancy childbirth* 2015;15(1):106.
13. Bhutta ZA, Soofi S, Cousens S, et al. Improvement of perinatal and newborn care in rural Pakistan through community-based strategies: a cluster-randomised effectiveness trial. *The Lancet* 2011;377(9763):403-12.
14. Soofi S, Cousens S, Imdad A, et al. Topical application of chlorhexidine to neonatal umbilical cords for prevention of omphalitis and neonatal mortality in a rural district of Pakistan: a community-based, cluster-randomised trial. *The Lancet* 2012;379(9820):1029-36.
15. Habib MA, Soofi S, Sadiq K, et al. A study to evaluate the acceptability, feasibility and impact of packaged interventions ("Diarrhea Pack") for prevention and treatment of childhood diarrhea in rural Pakistan. *J BMC Public Health* 2013;13(1):922.
16. Bari A, Sadruddin S, Khan A, et al. Community case management of severe pneumonia with oral amoxicillin in children aged 2–59 months in Haripur district, Pakistan: a cluster randomised trial. *The Lancet* 2011;378(9805):1796-803.
17. Soofi S, Cousens S, Turab A, et al. Effect of provision of home-based curative health services by public sector health-care providers on neonatal survival: a community-based cluster-randomised trial in rural Pakistan. 2017;5(8):e796-e806.

For peer review only

18. Rabbani F, Mukhi AAA, Perveen S, et al. Improving community case management of diarrhoea and pneumonia in district Badin, Pakistan through a cluster randomised study—the NIGRAAN trial protocol. 2014;9(1):186.
19. National Institute of Population Studies Islamabad Pakistan. Pakistan Demographic and Health Survey 2017-2018. Pakistan: National Institute of Population Studies Islamabad Pakistan,, 2019.
20. National Institute of Population Studies Islamabad Pakistan. Pakistan Demographic and Health Survey 2012-2013. Pakistan, 2013.
21. jhpiego. Training and Programming Materials. Explore the tools you need. 2019 [Available from: https://hms.jhpiego.org/training-materials/.
22. American Academy of Paediatrics. Helping Baby Survive 2019 [Available from: https://www.aap.org/en-us/advocacy-and-policy/aap-health-initiatives/helping-babies-survive/Pages/default.aspx
23. World Health Organisation. GAPPD: ending preventable child deaths from pneumonia and diarrhoea by 2025 2019 [Available from: https://www.who.int/woman_child_accountability/news/gappd_2013/en/.
24. Zaidi S, Bigdeli M, Aleem N, et al. Access to essential medicines in Pakistan: policy and health systems research concerns. *PloS one* 2013;8(5):e63515.
25. Zhu N, Allen E, Kearns A, et al. Lady health workers in Pakistan: improving access to health care for rural women and families, 2014.
26. Nishtar S, Boerma T, Amjad S, et al. Pakistan's health system: performance and prospects after the 18th Constitutional Amendment. *The Lancet* 2013;381(9884):2193-206.
27. Mphatswe W, Mate KS, Bennett B, et al. Improving public health information: a data quality intervention in KwaZulu-Natal, South Africa. 2012;90:176-82.

For peer review only - http://bmjopen.bmj.com/site/about/guidelines.xhtml

Figure 1: Project design of Umeed-e-Nau

Figure 2: Umeed-e-Nau Project Timeline

Figure 3: Map showing the geographic location of the program districts in three provinces of Pakistan

Figure 4: Schema of intervention packages for the implementation of maternal and newborn interventions in Pakistan

For peer review only

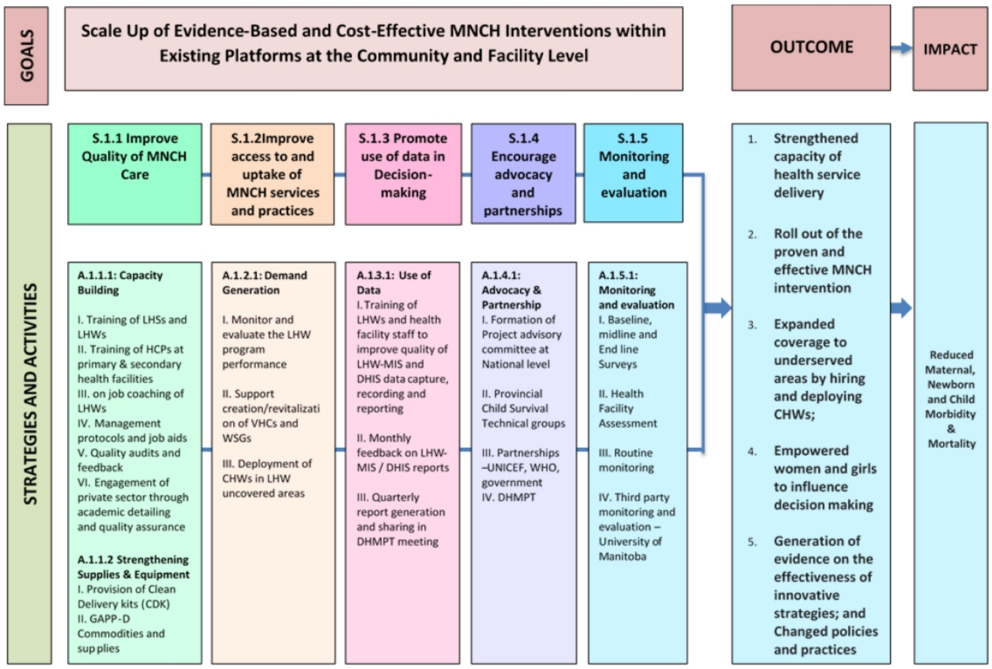

Project design of Umeed-e-Nau

244x164mm (300 x 300 DPI)

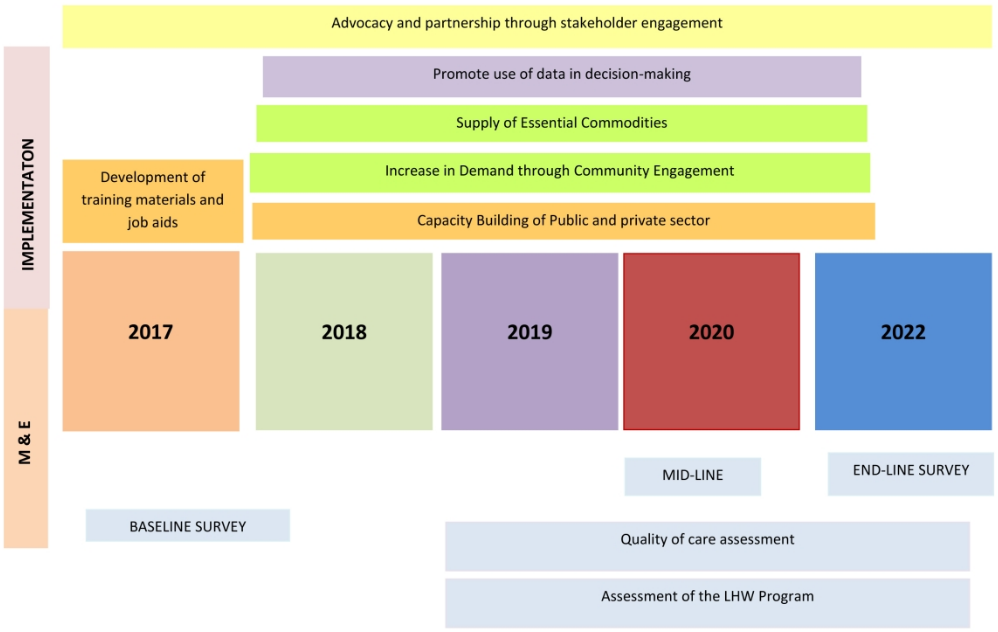

Umeed-e-Nau Project Timeline

220x142mm (300 x 300 DPI)

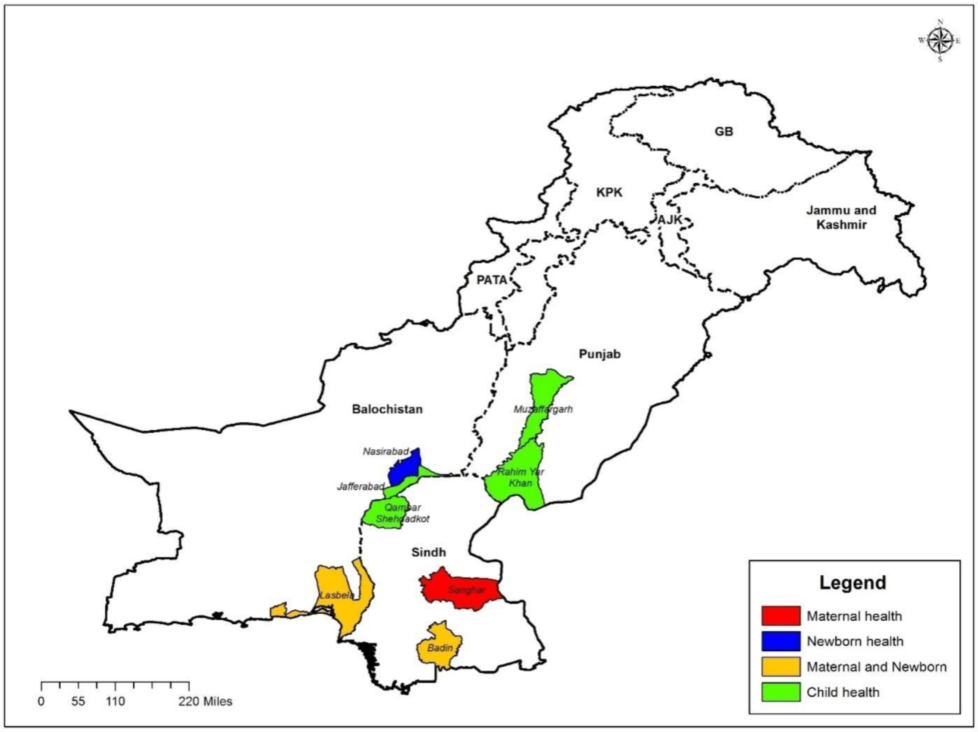

Map showing the geographic location of the program districts in three provinces of Pakistan

166x126mm (300 x 300 DPI)

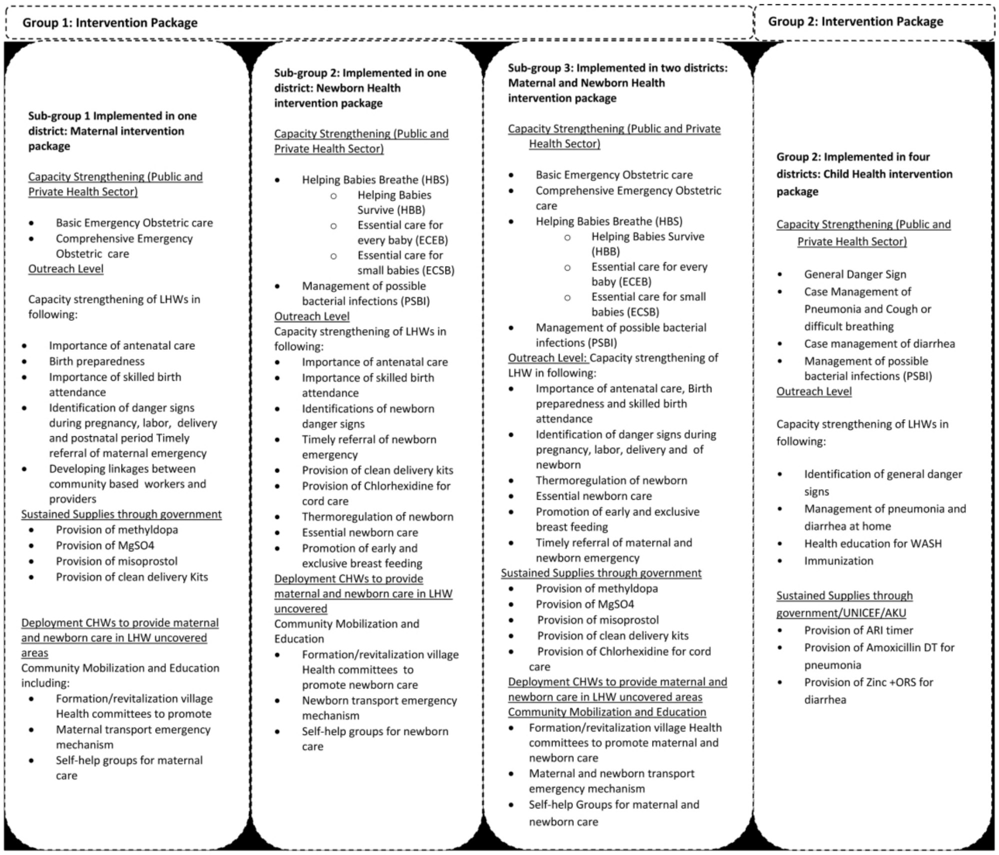

Schema of intervention packages for the implementation of maternal and newborn interventions in Pakistan

219x186mm (300 x 300 DPI)

BMJ Open

Effect and feasibility of district level scale up of maternal, newborn and child health interventions in Pakistan: a quasi-experimental study

Journal:	*BMJ Open*
Manuscript ID	bmjopen-2019-036293.R1
Article Type:	Protocol
Date Submitted by the Author:	18-Mar-2020
Complete List of Authors:	Memon, Zahid Ali; Aga Khan University, Centre of Excellence in Women and Child Health<br>Muhammad , Shah; Aga Khan University, Centre of Excellence in Women and Child Health<br>Soofi, Sajid; Aga Khan University Hospital, Center of Excellence in Women and Child Health<br>Khan , Nimra ; Aga Khan University, Centre of Excellence in Women and Child Health<br>Akseer, Nadia; The Hospital for Sick Children , Centre for Global Child Health<br>Habib, Atif; Aga Khan University, Center of Excellence in Women and Child Health<br>Bhutta, Zulfiqar; Aga Khan University, Center of Excellence in Women and Child Health; The Hospital for Sick Children , Centre for Global Child Health
**Primary Subject Heading**:	Public health
Secondary Subject Heading:	Evidence based practice
Keywords:	Community child health < PAEDIATRICS, Quality in health care < HEALTH SERVICES ADMINISTRATION & MANAGEMENT, PUBLIC HEALTH

SCHOLARONE™
Manuscripts

I, the Submitting Author has the right to grant and does grant on behalf of all authors of the Work (as defined in the below author licence), an exclusive licence and/or a non-exclusive licence for contributions from authors who are: i) UK Crown employees; ii) where BMJ has agreed a CC-BY licence shall apply, and/or iii) in accordance with the terms applicable for US Federal Government officers or employees acting as part of their official duties; on a worldwide, perpetual, irrevocable, royalty-free basis to BMJ Publishing Group Ltd ("BMJ") its licensees and where the relevant Journal is co-owned by BMJ to the co-owners of the Journal, to publish the Work in this journal and any other BMJ products and to exploit all rights, as set out in our licence.

The Submitting Author accepts and understands that any supply made under these terms is made by BMJ to the Submitting Author unless you are acting as an employee on behalf of your employer or a postgraduate student of an affiliated institution which is paying any applicable article publishing charge ("APC") for Open Access articles. Where the Submitting Author wishes to make the Work available on an Open Access basis (and intends to pay the relevant APC), the terms of reuse of such Open Access shall be governed by a Creative Commons licence – details of these licences and which Creative Commons licence will apply to this Work are set out in our licence referred to above.

Other than as permitted in any relevant BMJ Author's Self Archiving Policies, I confirm this Work has not been accepted for publication elsewhere, is not being considered for publication elsewhere and does not duplicate material already published. I confirm all authors consent to publication of this Work and authorise the granting of this licence.

For peer review only

Effect and feasibility of district level scale up of maternal, newborn and child health interventions in Pakistan: a quasi-experimental study

Zahid A Memon, MPH [1]; Shah Muhammad, MPH [1]; Sajid B Soofi, FCPS[1] Nimra Khan, MPH [1]; Nadia Akseer PhD [2]; Atif Habib, PhD[1]; Zulfiqar A Bhutta PhD [1, 2]

[1]Center of Excellence in Women and Child Health, Agha Khan University, Karachi, Pakistan
[2] Centre for Global Child Health, The Hospital for Sick Children, Toronto, ON, Canada

Corresponding Author:
Zulfiqar A. Bhutta
Qualification: FRCPCH, PhD
Founding Director, Center of Excellence in Women and Child Health
Aga Khan University, zulfiqar.bhutta@aku.edu
+92214930051 ext. 4782
Centre for Global Child Health, The Hospital for Sick Children, 686 Bay St, Ste 11.9801, Toronto, ON M5G 0A4, Canada (zulfiqar.bhutta@sickkids.ca).

For peer review only

ABSTRACT

Introduction

Pakistan has a high burden of maternal, newborn and child morbidity and mortality. Several factors including weak scale-up of evidence-based interventions within the existing health system; lack of community awareness regarding health conditions; and poverty contribute to poor outcomes. Deaths and morbidity are largely preventable if a combination of community and facility-based interventions are rolled out at scale.

Methods and analysis

Umeed-e-Nau (UeN) (New Hope) project aims is to improve maternal, newborn and child health (MNCH) in 8 high burden districts of Pakistan by scaling up of evidence-based interventions. The project will assess interventions focused on firstly, improving the quality of MNCH care at primary and secondary levels. Secondly, interventions targeting demand generation such as community mobilization, creating awareness of healthy practices and expanding coverage of outreach services will be evaluated. Thirdly, we will also evaluate interventions targeting the improvement in quality of routine health information and promotion of use of the data for decision making. Hypothesis of the project is that roll out of evidence based interventions at scale will lead to at least 20% reduction in perinatal mortality and 30% decrease in diarrhea and pneumonia case fatality in the target districts whereas two intervention groups will serve as internal controls.

Monitoring and evaluation of the program will be undertaken through conducting periodical population level surveys and quality of care assessments. Descriptive and multivariate analytical methods will be used for assessing the association between different factors and difference in difference estimates will be used to assess the impact of the intervention on outcomes.

Ethics and Dissemination

The ethics approval was obtained from the Aga Khan University Ethics Review Committee (ERC).The findings of the project will be shared with relevant stakeholders and disseminated through open access peer reviewed journal articles.

This trail is registered, NCT04184544.

Key Words

For peer review only

Maternal, Neonatal, Child Health, Interventions, Mortality, Community child health, Public Health, Quality of care

Word Count 3669

Strengths and limitations of this study

- Strength of the UeN project includes its quasi experimental study design with district representative sample size
- The household surveys will be powered to measure district level perinatal and child mortality estimates
- Interventions to promote use of data for decision making will be assessed in Pakistan for the first time
- Potential limitations include challenges in accessing routine service delivery data for evaluation purposes
- Challenges of working with or assessing role of LHWs who are already overburdened with competing priorities

For peer review only

INTRODUCTION

Pakistan's high maternal, neonatal, and child mortality continues to hamper its overall growth and development.[1] The global maternal mortality ratio in 2017 is estimated at 211 maternal deaths per 100 000 live births while 2019 estimates by United Nations inter-agency group for child mortality estimation (UN-IGME) estimate neonatal mortality rate (NMR) at 18 deaths per 1000 live births and under-5 mortality rate is 39 per 1,000 live births per year globally.[2,3] Diarrhea and pneumonia are two of the most common causes of child mortality; together, they account for more than 30% of all under-5 deaths in Pakistan.[4]

Several factors have contributed to the slow progress in maternal, newborn, and child health (MNCH) in Pakistan, including social determinants such as poverty, high rates of illiteracy, lack of women's and girls' empowerment, voice, and high fertility rates. These are compounded by poorly functional health systems with low coverage rates of essential health interventions, wide disparities by geographic and socio-economic status and a largely unregulated private sector providing poor quality health services;[5] and lack of community awareness and timely care seeking for health conditions.[6,7,8] The majority of maternal, newborn and child deaths are preventable and treatable given programs are designed and delivered through an efficient public and private health system that takes into account the geographic and social structures.[9]

The health system and services operate through both public and private sectors in Pakistan. The public sector features a three-tiered health delivery system offering a range of essential public health interventions. The basic health units (BHUs) and rural health centers (RHCs) together with the community outreach program i.e. the Lady Health Worker (LHW) program provide core primary healthcare services; tehsil headquarter hospitals (THQs) and district headquarter hospitals (DHQs) serve as first and second level referral facilities with ambulatory and inpatient care.[10]

An LHW is associated with the public health facility usually at primary and secondary level. Each LHW has a catchment population comprising of approximately 200 households.[11] They target specific groups such as families with children under 5 and married couple eligible for family planning. Their key functions include increasing awareness about contraception, MNCH and nutrition. They also provide treatment for common illnesses like Diarrhea and acute respiratory infections (ARI) and distribute contraceptives for family planning. Furthermore, they identify danger signs for illnesses and refer people from the community to the health facility.

For peer review only

Around 70% of the population uses private providers, with expenses often paid out of pocket. Private providers are largely unregulated, though provincial governments have recently set-up regulatory health care commissions to reduce high levels of unqualified providers and initiated licensing of private facilitie.[1]

A number of strategies have been employed at community and facility levels to strengthen the public health service delivery system in Pakistan and elsewhere to improve the MNCH indicators.[12] Such approaches have attempted to improve the quality of care through reviewing and updating training material[13]; conducting trainings and providing essential commodities to healthcare providers and community health workers[14].[15] For example, job aids led intervention to improve quality of antenatal counselling in the Zou/Collines regions of Benin showed positive impact on danger sign recognition, birth preparedness, clean delivery and newborn care .[16] Training of healthcare providers (HCPs) improved contact and quality across the continuum of care in Ghana[17]. Furthermore, capacity building initiatives for health care workers have resulted in improved care quality of MNCH services in Jigawa State, northern Nigeria[18]. Provision of job aids and training of LHWs in Gilgit region of Pakistan had a significant reduction of 38% in perinatal mortality rate.[19] Furthermore, birth kits with 4% chlorhexidine for application to the umbilical cord were provided to traditional birth attendants to prevent omphalitis and thereby effecting neonatal mortality resulting in 38% reduction in neonatal mortality.[20] A diarrhea pack with oral rehydration salts (ORS) and Amoxicillin distributed was feasible and acceptable with an impact on the incidence of diarrhea and reduced hospitalization rates.[21]

Strategies to improve MNCH have also focused on improving demand and supply side factors through community mobilization activities and setting up of self-help groups. Formation of community groups in Magu district, Tanzania to identify and resolve issues during pregnancy and childbirth resulted in an increased maternal health knowledge and positive behavior changes among health care workers and community.[22] A behavior change intervention with women's self-help group was evaluated in rural India.[23] They found significant improvement in using contraceptive methods, had institutional delivery, practiced skin-to-skin care, initiated timely breastfeeding, exclusively breastfed the child, and provided age-appropriate immunization. Furthermore, community mobilization intervention led by LHWs in Hala, Pakistan resulted in 21% reduction of in stillbirth rate and 15% neonatal mortality rate over two years period.[24] Community management of pneumonia through LHWs showed LHWs can successfully diagnose and treat severe pneumonia at home. The LHWs were able to effectively reach children with pneumonia where referral was difficult.[15] Community case management of pneumonia through LHWs

For peer review only - http://bmjopen.bmj.com/site/about/guidelines.xhtml

with oral amoxicillin at home was tested in two districts of Pakistan and was proven to be safe and effective for treatment of severe pneumonia through LHWs.[25]

Furthermore, there is evidence on interventions for strengthening health information system.[26]For instance a simple data improvement intervention led to completeness and accuracy of mother-to-child transmission of the human immunodeficiency virus data in KwaZulu-Natal, South Africa.[27]

Lastly, it is evident from previous studies that scaling up interventions using delivery platforms to reach poor and rural populations through community-based strategies and their successful implementation can prevent 58% of an estimated 367 900 deaths (15 900 maternal, 169 000 newborn, 183 000 child deaths) and 49% of an estimated 180 000 stillbirths in Pakistan alone.[15,19,24, 20, 21, 26,28]

The UeN initiative aims to improve the health, well-being and survival of women and children in Pakistan through piloting and implementing effective MNCH interventions at scale in 8 high burden districts spanning 3 provinces and targeted to reach more than 11 million beneficiaries. The UeN initiative's main goal is to scale up the evidence-based and cost-effective MNCH interventions within existing platforms at the community and facility level. Study goal, objectives and activities are detailed in Figure 1. It is anticipated the UeN intervention scale up in 8 districts will reach approximately 1.7 million under-5 children, 1.5 million women of reproductive age, and 8.6 million other civilians. The intervention will target 509 public health facilities (19 maternal and child health centers, 150 Government dispensaries, 255BHUs, 59 RHCs, 18 THQs 6 DHQs, 2 civil hospitals), 500 private providers, 6518 LHWs, and 300 CHWs. The primary hypothesis of the study is to assess whether roll out of proven and effective MNCH interventions at scale using existing public and private healthcare platforms at primary and secondary care level can reduce perinatal deaths by 20% and 30% reduction in diarrhea and pneumonia case fatality rates.

For peer review only

Objectives

1. Improve quality of MNCH care through development of training material and job aids for healthcare workforce; capacity building of healthcare workers; engaging private sector HCPs and to ensure supply of essential commodities at community and facility level.
2. Improve access to and uptake of MNCH services through targeting supply and demand side factors. The focus will be to create awareness on healthy practices at household level; revitalizing and creating village health committees; expanding community based services to areas, which are currently not covered.
3. Promotion of the use of data for decision-making. Interventions will be used to improve data quality of public health facility and community level information systems. Furthermore, information system will be established for private healthcare providers.

This protocol paper will present the design and roll-out strategy for the UeN initiative. We will outline the setting, methods, implementation strategies and monitoring & evaluation processes of the project roll-out spanning years 2017-2022 (project timeline shown in Figure 2).

METHODS AND ANALYSIS

Study setting

Covering 3 provinces in the country, the UeN scale up component will be implemented in 8 districts (location of interventions by theme is shown in Figure 3). Study districts were selected to be predominantly rural, and had some of the highest MNCH morbidity and mortality outcomes in the country. An important consideration was also ensuring that selected districts did not have other major public health campaigns to avoid conflation or dilution of UeN impact. High burden districts were strategically selected to demonstrate effective change models in difficult geographies so as to maximize project impact on population outcomes. Table 1 [29] outlines relevant demographic, health and contextual indicators for each study district.

Table 1: Distribution of demographic and health indicators for maternal, newborn and child health indicators in districts selected for program implementation in UeN initiative in Pakistan

	**Maternal and Newborn Health**	**Maternal health**	**Newborn Health**	**Child Health**

For peer review only

	Lasbela	Badin	Sanghar	Nasirabad	Qambar Shahdadkot	Muzaffargarh	Jafferabad	Rahim Yar Khan
**Population**	574,292	1,804,516	2,057,057	490,538	1,341,042	4,325,483	513,813	4,814,006
**U5 population**	74,657	202,106	230,390	13,837	150,197	462,756	14,191	625,8208
**Human development index (HDI)**	0.416	0.412	0.491	0.311	0.456	0.584	0.345	0.625
**HDI rank**	85	87	72	98	77	58	94	51
**Tehsils/Talukas**	5	5	6	4	7	4	3	4
**Union Councils**	22	12	63	31	52	93	38	139
**Vaccination coverage- Children 12-23 months (fully immunized)**	28.8	45.5	47.4	28.8	25.6	82.1	28.8	72.0
**ANC during last pregnancy (%)**	55.9	83.1	56.8	55.9	64.5	37.4	55.9	30.8
**Facility births (%)**	34.6	64.8	40.0	34.6	48.7	50.7	34.6	62.0
**Delivery by Skilled Birth Attendant**	38.2	66.6	42.1	38.2	49.7	52.7	38.2	65.6
**Postnatal consultation in Health Facility**	29	75.0	61.6	29	68.2	56.9	29	63.9
**Access to tap water**	29.6	20.7	20.7	28.6	5.8	6.5	28.6	21.4
**Access to toilet (Flush)**	17.6	70	51.8	16.5	77.3	72	16.5	60.5

For peer review only

**Number of LHWs**	153	1010	883	309	680	1891	130	1723
**LHWs coverage %**	50	80	66	60	59	51	50	80
**Number of Hospitals**	125	145	116	97	271	248	97	260
**Number of RHCs**	4	12	5	1	4	12	1	19
**Number of BHUs**	42	36	58	19	4	76	19	103

Study design

In order to evaluate the impact of scaling up MNCH interventions, a quasi-experimental study design will be used. Each of the 8 districts will be treated as an intervention site, and will be grouped together broadly into two areas to span MNCH interventions (Figure 3). As detailed below, group 1 will receive maternal and newborn health (MNH) interventions, while group 2 will receive child health (CH) interventions. Each group will serve as the "control" for the other. This design will help us evaluate independent and combined effect of maternal, newborn, and child health interventions to address MNCH outcomes.

Site allocation to Group1 and 2 were done through extensive consultation with provincial government, bilateral donors and UN agencies, and UeN project team. Factors that were considered during intervention allocation included level of MNCH morbidity and mortality, assuring geographical diversity across the provinces, varying size of the districts, and government MNCH priorities in those districts (i.e. so that those areas that were focused on improving CH, for example, received the CH interventions). While this design might have ethical implications such as not providing all MNCH related evidence based interventions to the entire population however population in control areas will continue to receive all the routine services which are part of the public sector standard of care and some additional MNCH intervention as per allocation to the type of intervention group. Furthermore, this ethical concern has been addressed by developing partnerships with Government, development

For peer review only

agencies and private providers who have agreed to include UeN interventions in the existing health system, if the evidence generated suggests their effectiveness and feasibility when delivered at scale.

Sample size

The sample size for the household surveys will be calculated using the assumption that that the roll out of proven and effective interventions within existing health delivery platforms for MNCH will lead to significant reduction in perinatal mortality rate (20% compared to baseline) and at least 30% reduction in diarrhea and pneumonia case fatality rates in the target districts for the proposed UeN scale-up initiative. Perinatal mortality (PNM) will be used as the primary outcome for all sample size projections. A stratified two stage cluster sampling approach will be used to calculate sample size. Moreover, it is important to power the study to provide a detectable difference between the two groups of Maternal and Newborn health group, and the Child Health group. We will use a before and after design for sample size calculation. For maternal and newborn health districts, assuming a 20% reduction in the PNM from the baseline PNM of 75 per 1000 live births (Pakistan Demographic Health Survey 2012-13 for rural Pakistan)[30] with a power of 80% to detect the difference in the PNM between baseline, and the end line household survey, a type I error of 5%, design effect of 1.515 and an annual attrition of 10% per year, at least 19,582 households will be enrolled in the survey. For Child Health intervention districts, assuming a 30% reduction in the Post Neonatal Death (PND) rate of 19/1000 live births at baseline (Pakistan Demographic Health Survey 2012-13 for rural Pakistan) with a power of 80% to detect the difference in the PND between baseline, and the end line household survey, a type I error of 5%, design effect of 1.388 and an annual attrition of 10% per year, at least 19,210 households have to be enrolled in the survey.

Intervention packages and implementation strategies

An integrated intervention package, that has shown to be relevant, feasible, and effective is designed thematically (Figure 4) and will be delivered at scale in the target districts. Group 1: Maternal Health: These 4 districts (Badin, Sanghar, Lasbela, Nasirabad) will receive core MNH interventions as included in global recommendations for maternal health (Helping Mothers Survive)[31] and newborns (Helping Babies Survive)[32] consisting of three modules: Helping Babies Breathe, Essential Care for Every Baby, and Essential Care for Small Babies. We stratified intervention delivery into 3 sub-groups focused separately on maternal health (1 district: Sanghar); newborn health (1 district: Nasirabad); and combined maternal and newborn health (two districts: Lasbela and Badin). This stratification was undertaken to independently evaluate the relative impact of theme-specific interventions on key MNH and mortality outcome indicators; i.e. akin to a 3-arm trial design.

For peer review only - http://bmjopen.bmj.com/site/about/guidelines.xhtml

Group 2: Child Health: These 4 districts (Qambar Shahdadkot, Muzaffargarh, Rahim Yar Khan and Jafferabad) representing 3 provinces will receive child health interventions focusing on the implementation of the Global Action Plan for Pneumonia and Diarrhea (GAPPD).[33]

Details on UeN intervention approaches packages are displayed in Figure 1 and are discussed below by each of the 5 objectives.

Objective 1: Improve Quality of MNCH Care

Activity 1.1. Development of training material and job aids

UeN will adopt and contextualize development of training material and job aids in accordance with global and national guidelines to build provider and health worker knowledge, skills, and practices. The project team along with experts from the academia and government programs will develop training packages (including trainer and learner materials, as well as supplemental job aids and guidance) for LHWs and for public and private HCPs.

For private HCPs, 14 modules for on-site learning using academic detailing to refresh the private providers' knowledge will be developed.

Activity 1.2. Capacity Building

UeN will employ a mix of capacity building activities to address both outreach workers' and public sector HCPs' performance and confidence in achieving quality standards. Training content will be based on the district's theme. Topics to be covered in the training are listed in Figure 4.

- *Outreach Health Workers Training: Lady Health Workers*

The LHWs' skills will be strengthened to promote healthy MNCH practices and behaviors at community-level and will be trained to adhere to care management protocols and use new commodities and technology which includes chlorhexidine, amoxicillin dispersible tablets (dt), zinc dt and low osmolality ORS and respiratory counters etc. The LHW training will be based on low dose high frequency model. [19,24]

- *Facility based: Training for providers at primary and secondary level public health facility*

Trainings for public sector HCPs will be group-based trainings where participants will gather in a public health care facility for a competency-based learning activity.

Activity 1.3. Private sector engagement

Private sector HCPs will be engaged through academic detailing, where individual health providers from private sector (or a facility-based team of providers) will participate in a structured learning activity at their facility. Topics on key areas of MNCH service delivery will be covered.

For peer review only - http://bmjopen.bmj.com/site/about/guidelines.xhtml

Initial interaction will be followed by facility-based coaching and mentoring.

Activity 1.4. Sustained key commodities and supplies by increasing efficiency of the supply chain for essential commodities at community and facility level

Stock out of MNCH commodities is a major bottleneck in delivering quality services within the health system.[34] LHWs, primary and secondary level health care facilities in the target districts will be equipped with essential drugs and equipment to provide quality services through engaging officials of the department of health and other stakeholders. The commodities and supplies will be ensured according to district themes such as for maternal and newborn health districts, clean delivery kit will be provided to LHWs serving in maternal and newborn health districts.

For the four MNH districts, clean delivery kits will be provided by Aga Khan University (AKU). For the CH districts, supplies of amoxicillin dt (250 mg), zinc dt, ARI timer and pulse oximeter will be provided by UNICEF in three districts of Punjab and Sindh province, however since the current UNICEF programme does not cover provision of commodities to Balochistan, AKU will supply the commodities to the child health district in Balochistan.

For peer review only

Objective 2: Improve access to and uptake of MNCH services and practices

Activity 2.1. Demand generation

- **Improve uptake of healthy practices at household level through community mobilization**

Through community mobilization activities the community will be informed through LHWs about danger signs during pregnancy, child birth, infancy and childhood. The importance of early referral by LHWs to health facilities for prevention of morbidity and mortality will be the focus of community mobilization activities.

- **Community engagement through revitalization/creation of women support groups/ village health committees**

LHWs will be trained to revitalize and create where required the self-help groups, village health committees and women support groups to discuss MNCH issues. These groups are expected to help women to adopt healthy MNCH practices.

Activity 2.2: Expanding the coverage to underserved areas

In current public health outreach system, LHWs provide services at the household level. However, only 50-60% of the rural districts are covered by LHWs.[35] To serve the geographic areas for which LHWs are not appointed, community health workers (CHWs) will be hired and will be trained on technical MNCH content, registration, community support group methodology and village health committee formation. Around 100 per district will be deployed. CHWs will be selected on the lower education qualification such as primary education or lower as compared to LHWs who are minimum middle pass. The CHW training will consist of lectures, interactive discussions, and role-plays to develop the skills of performing their roles. The duration of the training will be two weeks, twice in the first year, and then a refresher training every year. CHWs will only be recruited and deployed in districts that have maternal, newborn or combined component due to current scope of community health worker programs.

Objective 3: Promote use of data for decision-making

In Pakistan, there are two main sources of data for decision making the District Health Information System (DHIS) and the LHW-Management Information System (MIS) for health facility and community level information respectively. The private sector HCPs do not share routine service delivery data in the DHIS. To promote data use; UeN's objective is to target all three data sources. Initially, a gaps analysis will be conducted to identify the weaknesses in the system

Activity 3.1. Improving public health facility related information system i.e. DHIS

The routine service delivery data of both primary and secondary level health facilities is managed through DHIS system. The DHIS collects data on the 18 priority diseases, availability of drugs, service utilization

For peer review only

and human resource of health facilities. Data from each district is discussed in the quarterly district level meetings. However, there is poor compliance with the source registers which is the main source of information. As the data quality is poor and questionable, therefore it cannot guide decision making.[36]To improve understanding and importance of collecting data; HCPs of public health facilities will be trained on collection, recording and reporting of the data for DHIS.

For better compliance with source register and data quality, the project team will collect data from source registers on 40 key MNCH indicators every month. Data for these indicators will also be extracted from DHIS monthly reports from the online available dashboard. Values for the indicators from both source register and DHIS dashboard will be compared to assess data completeness and quality.[27] Monthly feedback report on data quality and completeness will be shared with the managers of health facilities and district managers. Furthermore, quarterly feedback report will be provided about the data quality and summary measures of key MNCH indicators in the district level management meetings.

Activity 3.2 Improving community based health information system i.e. LHW-MIS

The LHW is affiliated with a primary health facility and has a catchment population of 1000 and maintains their record e.g. number of married couple, pregnant women, under-5 children and service provision on source registers provided by the LHW program.[35] The information collected from the community level, is compiled on a monthly report and shared with their supervisors lady health supervisor (LHS) and then relayed to the district coordinator for the LHW Program. However, the data quality is not monitored regularly and data use for decision-making is minimal. To improve the data quality, the LHS and LHWs will be trained on the importance of accurate data recording and reporting. Furthermore, 10 LHWs will be randomly selected from their reporting health facility every month; data from their source registers will be collected on 29 MNCH related indicators on an android application. Values for the indicators from both source register and LHW-MIS dashboard will be compared to assess data completeness and quality. Monthly feedback reports will be shared with LHS and LHWs. Furthermore, quarterly feedback report about the data quality and summary of key MNCH indicators will be shared in the district level meetings.

Activity 3.3.Establishing Private Providers –Management Information System (MIS)

A private provider – management information system will be established. Routine data collection registers similar to the DHIS source registers with indicators according to the district themes will be developed and distributed to the private providers. Private providers will be encouraged to record data. The project team will collect data on monthly visits from the health facility. This will enable capture of MNCH related morbidities and mortalities which are otherwise lost due to non-reporting.

For peer review only - http://bmjopen.bmj.com/site/about/guidelines.xhtml

Monitoring and Impact Evaluation

UeN scale up will undertake rigorous monitoring and evaluation of core activities to ensure project progress and achievement of desired goals. Specific activities are detailed below:

- **Household surveys**

Household surveys will be conducted at three time points, baseline, at approximate mid-line when the interventions have been implemented at scale for at least 18-24 months, and at the end of the project (towards the end of year 5). The surveys will collect information to assess the impact of the interventions on primary and secondary outcome indicators (listed in table 2).

Table 2: List of primary and secondary outcomes

**Outcomes**	**Definition**
**Primary Outcomes**	
**Perinatal mortality rate**	The number of stillbirths and deaths in the first week of life per 1,000 total births
**Diarrhea case fatality**	Percent deaths among under-5 children due to diarrhea
**Pneumonia case fatality**	Percent deaths among under-5 children due to Pneumonia
**Secondary Outcomes**	
**Maternal and Newborn Health**	
**Contraceptive use**	Percent of mothers aged 15-49 who are using modern contraceptive method
**Antenatal care coverage**	Percent of mothers aged 15-49 of who received antenatal care ≥4 times during pregnancy with their youngest living child aged <6 months

For peer review only - http://bmjopen.bmj.com/site/about/guidelines.xhtml

**Tetanus Toxoid coverage**	Percent of mothers aged 15-49 who received ≥2 doses of tetanus toxoid during pregnancy with their youngest living child
**Skilled birth Attendant at time delivery**	Percent of mothers aged 15-49 whose youngest living child was delivered by skilled health personnel
**Institutional Delivery**	Percent of mothers aged 15-49 whose youngest living child was delivered in a health facility
**Postnatal care coverage**	Percent of mothers aged 15-49 who received postnatal care (for self or for infant) within 3 days of birth
**Early initiation of breastfeeding**	Percent of mothers aged 15-49 whose youngest living child was breastfed within one hour of birth
**Exclusive breastfeeding**	Percent of mothers aged 15-49 whose youngest living child was exclusively breastfed
**Vaccination coverage among under-5 children**	Percent of mothers aged 15-49 whose youngest living child received ≥3 doses of DPT/pentavalent vaccine when aged 12-23 months
	Percent of mothers aged 15-49 whose youngest living child received ≥1 dose of measles vaccine when aged 12-23 months
	Percent of mothers aged 15-49 whose youngest living child was fully immunized
**Care-seeking for Diarrhea among under-5 children**	Percent of mothers aged 15-49 whose youngest living child with diarrhea in the previous 2 weeks received ORS and zinc
**Care-seeking for Pneumonia among under-5 children**	Percent of mothers aged 15-49 whose youngest living child with pneumonia-like symptoms in the previous 2

For peer review only - http://bmjopen.bmj.com/site/about/guidelines.xhtml

	weeks was taken to an appropriate health provider and received antibiotics
**Capacity building**	Number of healthcare providers trained per 10 000 population
	Number of LHWs trained per 10 000 population

For peer review only

- **Quality of care assessments**

Health facility assessment will be conducted bi-annually to provide information on facility level performance and service quality including availability of commodities and supplies, human resource and key equipment as well as observation of HCPs will be done using quality of care checklists to assess the effectiveness of trainings provided. In case of inadequate availability of resources or sub-optimal quality of care provided, arrangements for supplies will be done by liaising with stakeholders and refresher trainings will be provided to HCPs. All public health facilities (509 including 19 Maternal and child health centers, 150 Government dispensaries, 255BHUs, 59 RHCs, 18 THQs 6 DHQs, 2 civil hospitals) will be included in the study. For private providers, all health practitioners who provide services according to district theme will mapped. Practitioners with high patient flow will be included in the study for quality assessment and needs based support will be ensured.

For peer review only - http://bmjopen.bmj.com/site/about/guidelines.xhtml

- **Assessment of the community outreach program (i.e. LHW program)**

The activities of LHWs will be monitored on regular basis to strengthen monitoring system of LHW program and improve LHW functionality. 30% health facilities will be selected on quarterly basis, 10 LHWs will be selected using purposive sampling from each health facility. Data will be recorded in mobile application from the LHW's source registers for community support groups and 10 randomly selected households (*khandans*). The data for community support groups will be validated from the community and households will be visited to validate the household level data.

Study outcome measures

The primary outcomes of interest are reduction in perinatal mortality rate (20% compared to baseline) and at least 30% reduction in Diarrhea and Pneumonia case fatality rates. The secondary outcomes include coverage of essential intervention coverage across the continuum of care and maternal, newborn and child morbidity rates (Table 2).

Analysis Plan

Data Management

A custom made mobile application will be designed to collect data using android handheld devices. The data collected in field will be stored and accessible real time at Aga Khan University server. Android based application will be designed such that it can monitor the location of the data points, time at which data was collected, and time taken to fill each form. This will enable the data management unit consisting of IT specialists and statisticians to monitor data collection activities and quality. Furthermore, data cleaning will be done by identifying errors and missing entries before any analysis begins.

Statistical Analysis

Basic frequencies will be calculated for demographic, social equity, primary and secondary outcomes of the study at baseline, midline and endline surveys. Furthermore, the baseline data will be analysed using multivariate analytical methods to assess the perinatal and child mortality estimates and its determinants.

The overall impact of the UeN project will be measured assuming parallel trends within the two MNH and CH groups, in the absence of any intervention, difference-in-difference estimates will be used to assess the impact of the intervention on outcomes. An interaction term between group (intervention/control) and survey wave (baseline/end-line) will be included in each model. All the analyses will be done using STATA.

Ethics and Dissemination

For peer review only

Ethics approval of the study was obtained from the Aga Khan University Ethics Review Committee on February 28th, 2017. In addition, ethical clearance was sought from National Bioethics committee (NBC), Pakistan.

Given the large scope of the UeN project, it is essential to garner support from and share progress and findings of the study with the stakeholders. Therefore, stakeholder engagement activities will be conducted at different levels to inform policy, community, and district health authorities.

- **Participation in provincial child survival technical groups**

For reducing Diarrhea and Pneumonia incidence, UNICEF has initiated the establishment of provincial child survival committees in which UeN will participate. There are also existing provincial MNCH committees where AKU will have a seat by virtue of existing projects and the current UeN project. The UeN project will set up a system of monthly meetings with UNICEF and will share the project's progress and activties.

- **Formation of Project Advisory Committee at National level**

Moreover, National ministry of health services regulation and coordination will be requested to constitute a project advisory committee to help guide the UeN project through the existing federal ministry of health services and regulation to ensure coordination. The committee will meet once during the inception phase to discuss the project structure and remit, followed by bi-annual progress reviews to address discuss challenges in implementation and address with appropriate actions.

- **Patient and Public Involvement:**

The public was not involved in the design of the research tools but they will be part of the study and their feedback will be sought regarding intervention acceptability. The key findings will be shared with their representatives as part of the dissemination plan at local level.

Authors' contributions:

ZAB conceived the project and provided the technical and intellectual inputs as principal investigator in writing this manuscript and approved for submission. ZM produced the first draft and subsequent drafts of the paper. NA, NK, SM,AH and SS were involved in review and provided inputs on various aspects of the manuscript. All authors reviewed and approved various drafts and the final paper.

Funding statement: This work is supported by Bill and Melinda Gates Foundation grant number [OPP1148892].

For peer review only - http://bmjopen.bmj.com/site/about/guidelines.xhtml

Acknowledgments: We thank Rahat Qureshi, Shabina Arif, Ali Turab and their respective teams for their support in designing the intervention packages around maternal, newborn and child health.

Competing interests: The authors declare that they have no competing interests.

For peer review only

References

1. Bhutta ZA, Hafeez A. What can Pakistan do to address maternal and child health over the next decade? *J Health research policy and systems* 2015;13(1):S49.
2. World Health Organisation. Trends in Maternal Mortality: 1990 to 2019 Estimates by WHO, UNICEF, UNFPA, World Bank Group and the United Nations Population Division. Geneva: World Health Organisation, 2019.
3. United Nations inter-agency Group. Levels & Trends in Child Mortality Report 2019: Estimates Developed by the UN Inter-agency Group for Child Mortality Estimation, 2019.
4. Bhutta ZA, Hafeez A, Rizvi A, et al. Reproductive, maternal, newborn, and child health in Pakistan: challenges and opportunities. *The Lancet* 2013;381(9884):2207-18.
5. Shaikh BT. Private sector in health care delivery: a reality and a challenge in Pakistan. *Journal of Ayub Medical College Abbottabad* 2015;27(2):496-98.
6. Kassebaum NJ, Bertozzi-Villa A, Coggeshall MS, et al. Global, regional, and national levels and causes of maternal mortality during 1990–2013: a systematic analysis for the Global Burden of Disease Study 2013. *The Lancet* 2014;384(9947):980-1004.
7. World Health Organisation. WHO guidelines on preventing early pregnancy and poor reproductive outcomes among adolescents in developing countries Geneva: World Health Organisation 2011.
8. Khan NN, Puthussery S. Stakeholder perspectives on public-private partnership in health service delivery in Sindh province of Pakistan: a qualitative study. *J Public health* 2019;170:1-9.
9. Bhutta ZA, Cabral S, Chan Cw, et al. Reducing maternal, newborn, and infant mortality globally: an integrated action agenda. *J International Journal of Gynecology Obstetrics* 2012;119:S13-S17.
10. Bhutta ZA, Hafeez A, Rizvi A, et al. Reproductive, maternal, newborn, and child health in Pakistan: challenges and opportunities. 2013;381(9884):2207-18.
11. Zhu N, Allen E, Kearns A, et al. Lady Health Workers in Pakistan
Improving access to health care for rural women and families 2014 [Available from: https://cdn2.sph.harvard.edu/wp-content/uploads/sites/32/2014/09/HSPH-Pakistan5.pdf.
12. Bhutta ZA, Black RE. Global maternal, newborn, and child health—so near and yet so far. *J New England Journal of Medicine* 2013;369(23):2226-35.
13. Tran NT, Portela A, de Bernis L, et al. Developing capacities of community health workers in sexual and reproductive, maternal, newborn, child, and adolescent health: a mapping and review of training resources. 2014;9(4)
14. Bhutta ZA, Ali S, Cousens S, et al. Interventions to address maternal, newborn, and child survival: what difference can integrated primary health care strategies make? 2008;372(9642):972-89.
15. Soofi S, Ahmed S, Fox MP, et al. Effectiveness of community case management of severe pneumonia with oral amoxicillin in children aged 2–59 months in Matiari district, rural Pakistan: a cluster-randomised controlled trial. *The Lancet* 2012;379(9817):729-37.
16. Jennings L, Yebadokpo AS, Affo J, et al. Antenatal counseling in maternal and newborn care: use of job aids to improve health worker performance and maternal understanding in Benin. 2010;10(1):75.
17. Okawa S, Gyapong M, Leslie H, et al. Effect of continuum-of-care intervention package on improving contacts and quality of maternal and newborn healthcare in Ghana: a cluster randomised controlled trial. 2019;9(9):e025347.

For peer review only - http://bmjopen.bmj.com/site/about/guidelines.xhtml

18. Okereke E, Tukur J, Aminu A, et al. An innovation for improving maternal, newborn and child health (MNCH) service delivery in Jigawa State, northern Nigeria: a qualitative study of stakeholders' perceptions about clinical mentoring. 2015;15(1):64.
19. Memon ZA, Khan GN, Soofi SB, et al. Impact of a community-based perinatal and newborn preventive care package on perinatal and neonatal mortality in a remote mountainous district in Northern Pakistan. *J BMC pregnancy childbirth* 2015;15(1):106.
20. Soofi S, Cousens S, Imdad A, et al. Topical application of chlorhexidine to neonatal umbilical cords for prevention of omphalitis and neonatal mortality in a rural district of Pakistan: a community-based, cluster-randomised trial. *The Lancet* 2012;379(9820):1029-36.
21. Habib MA, Soofi S, Sadiq K, et al. A study to evaluate the acceptability, feasibility and impact of packaged interventions ("Diarrhea Pack") for prevention and treatment of childhood diarrhea in rural Pakistan. *J BMC Public Health* 2013;13(1):922.
22. Solnes Miltenburg A, van Pelt S, de Bruin W, et al. Mobilizing community action to improve maternal health in a rural district in Tanzania: lessons learned from two years of community group activities. 2019;12(1):1621590.
23. Saggurti N, Atmavilas Y, Porwal A, et al. Effect of health intervention integration within women's self-help groups on collectivization and healthy practices around reproductive, maternal, neonatal and child health in rural India. 2018;13(8)
24. Bhutta ZA, Soofi S, Cousens S, et al. Improvement of perinatal and newborn care in rural Pakistan through community-based strategies: a cluster-randomised effectiveness trial. *The Lancet* 2011;377(9763):403-12.
25. Bari A, Sadruddin S, Khan A, et al. Community case management of severe pneumonia with oral amoxicillin in children aged 2–59 months in Haripur district, Pakistan: a cluster randomised trial. *The Lancet* 2011;378(9805):1796-803.
26. Soofi S, Cousens S, Turab A, et al. Effect of provision of home-based curative health services by public sector health-care providers on neonatal survival: a community-based cluster-randomised trial in rural Pakistan. 2017;5(8):e796-e806.
27. Mphatswe W, Mate KS, Bennett B, et al. Improving public health information: a data quality intervention in KwaZulu-Natal, South Africa. 2012;90:176-82.
28. Rabbani F, Mukhi AAA, Perveen S, et al. Improving community case management of diarrhoea and pneumonia in district Badin, Pakistan through a cluster randomised study—the NIGRAAN trial protocol. 2014;9(1):186.
29. National Institute of Population Studies Islamabad Pakistan. Pakistan Demographic and Health Survey 2017-2018. Pakistan: National Institute of Population Studies Islamabad Pakistan,, 2019.
30. National Institute of Population Studies Islamabad Pakistan. Pakistan Demographic and Health Survey 2012-2013. Pakistan, 2013.
31. jhpiego. Training and Programming Materials. Explore the tools you need. 2019 [Available from: https://hms.jhpiego.org/training-materials/.
32. American Academy of Paediatrics. Helping Baby Survive 2019 [Available from: https://www.aap.org/en-us/advocacy-and-policy/aap-health-initiatives/helping-babies-survive/Pages/default.aspx
33. World Health Organisation. GAPPD: ending preventable child deaths from pneumonia and diarrhoea by 2025 2019 [Available from: https://www.who.int/woman_child_accountability/news/gappd_2013/en/.
34. Zaidi S, Bigdeli M, Aleem N, et al. Access to essential medicines in Pakistan: policy and health systems research concerns. *PloS one* 2013;8(5):e63515.

For peer review only - http://bmjopen.bmj.com/site/about/guidelines.xhtml

35. Zhu N, Allen E, Kearns A, et al. Lady health workers in Pakistan: improving access to health care for rural women and families, 2014.
36. Nishtar S, Boerma T, Amjad S, et al. Pakistan's health system: performance and prospects after the 18th Constitutional Amendment. *The Lancet* 2013;381(9884):2193-206.

For peer review only

Figure 1: Project design of Umeed-e-Nau

Figure 2: Umeed-e-Nau Project Timeline

Figure 3: Map showing the geographic location of the program districts in three provinces of Pakistan

Figure 4: Schema of intervention packages for the implementation of maternal and newborn interventions in Pakistan

For peer review only

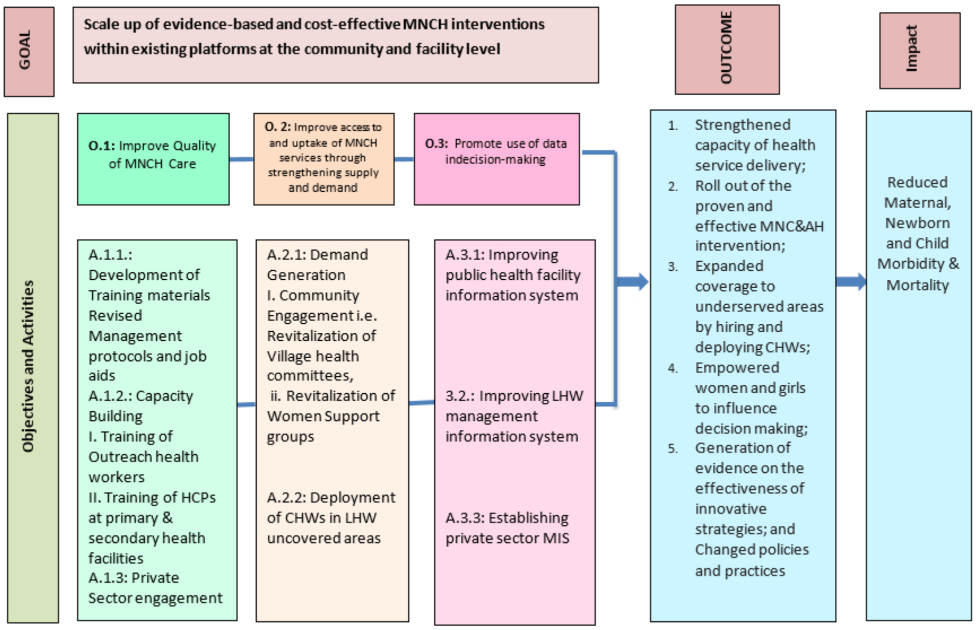

Project design of Umeed-e-Nau

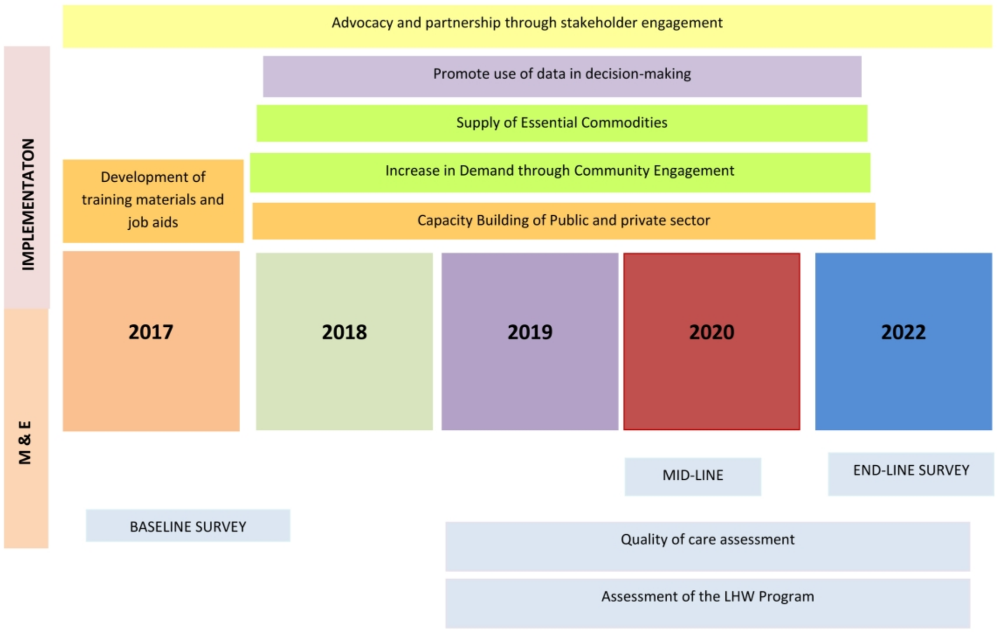

Umeed-e-Nau Project Timeline

220x142mm (300 x 300 DPI)

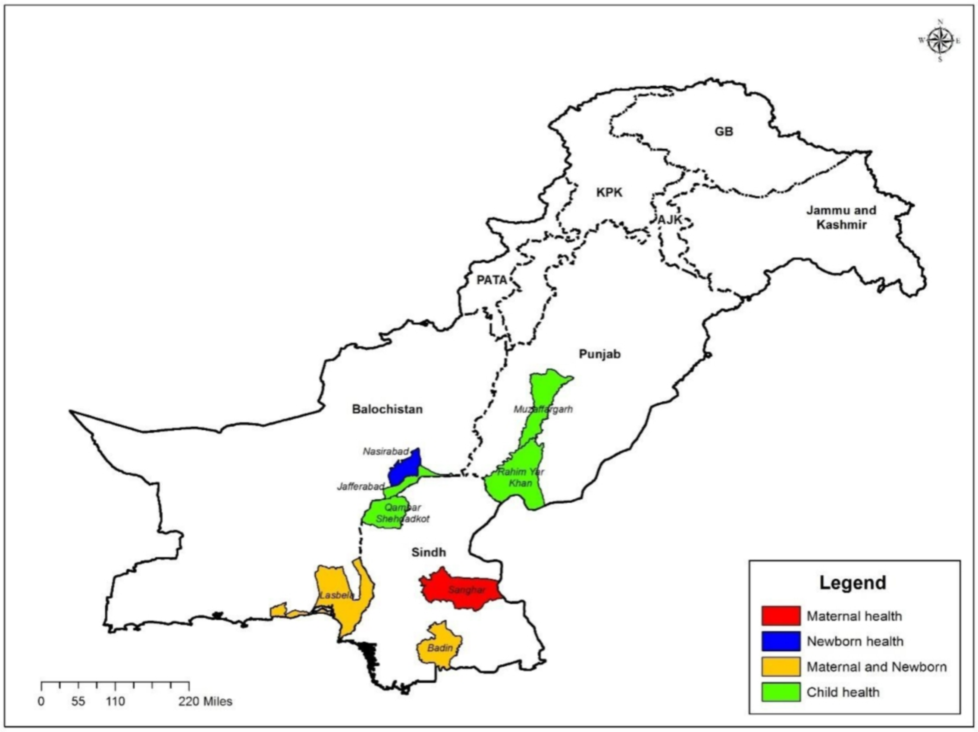

Map showing the geographic location of the program districts in three provinces of Pakistan

166x126mm (300 x 300 DPI)

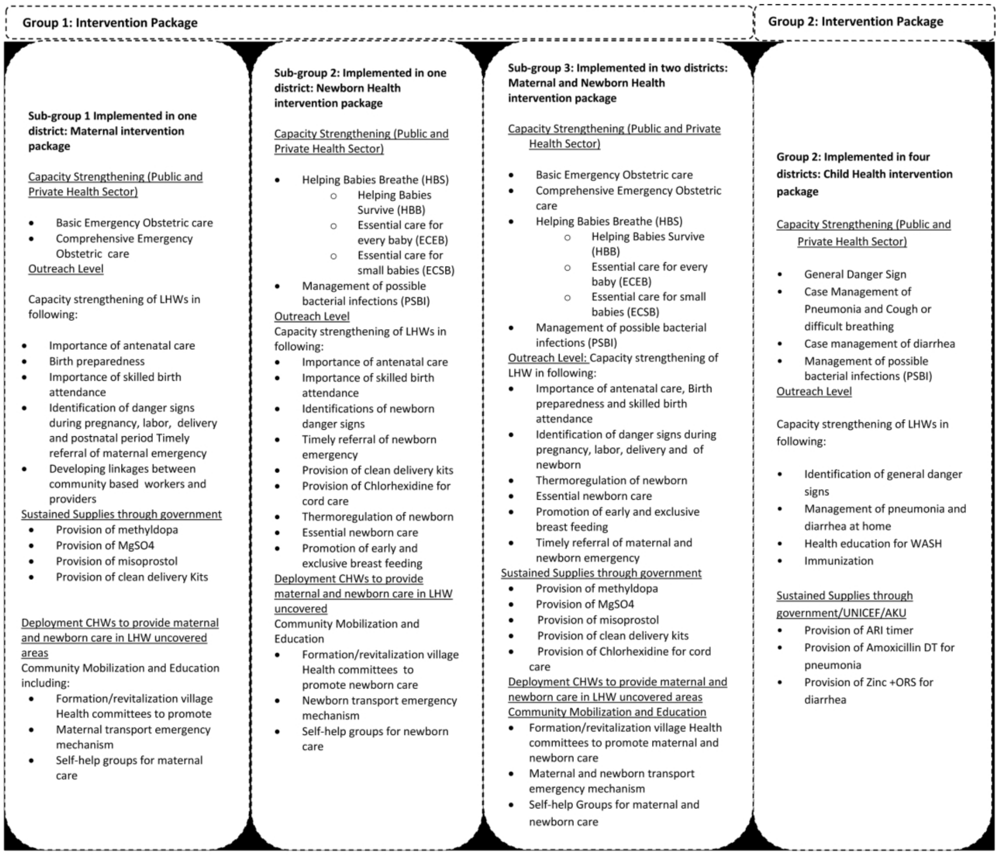

Schema of intervention packages for the implementation of maternal and newborn interventions in Pakistan

219x186mm (300 x 300 DPI)
